# Structural characterization of substrate and inhibitor binding to farnesyl pyrophosphate synthase from *Pseudomonas aeruginosa*

**DOI:** 10.1107/S1399004715001121

**Published:** 2015-02-26

**Authors:** Jason W. Schmidberger, Robert Schnell, Gunter Schneider

**Affiliations:** a Department of Medical Biochemistry and Biophysics, Karolinska Institutet, SE-171 77 Stockholm, Sweden

**Keywords:** *Pseudomonas aeruginosa*, farnesyl pyrophosphate synthase, ligand complexes, enzyme inhibitor, enzyme assay, isoprenoid biosynthesis

## Abstract

Locus PA4043 in the genome of *Pseudomonas aeruginosa* PAO1 has been annotated as coding for a farnesyl pyrophosphate synthase (FPPS). This open reading frame was cloned and expressed recombinantly in *Escherichia coli*. The dimeric enzyme shows farnesyl pyrophosphate synthase activity and is strongly inhibited by ibandronate and zoledronate, drugs that are presently in clinical use. The structures of the unliganded enzyme and complexes with the substrate geranyl diphosphate (GPP), the inhibitor ibandronate and two compounds obtained from a differential scanning fluorimetry-based screen of a fragment library were determined by X-ray crystallography to resolutions of better than 2.0 Å. The enzyme shows the typical α-helical fold of farnesyl pyrophosphate synthases. The substrate GPP binds in the S1 substrate site in an open conformation of the enzyme. In the enzyme–ibandronate complex three inhibitor molecules are bound in the active site of the enzyme. One inhibitor molecule occupies the allylic substrate site (S1) of each subunit, as observed in complexes of nitrogen-containing bisphosphonate inhibitors of farnesyl synthases from other species. Two (in subunit *A*) and one (in subunit *B*) additional ibandronate molecules are bound in the active site. The structures of the fragment complexes show two molecules bound in a hydrophobic pocket adjacent to the active site. This allosteric pocket, which has previously only been described for FPPS from eukaryotic organisms, is thus also present in enzymes from pathogenic prokaryotes and might be utilized for the design of inhibitors of bacterial FPPS with a different chemical scaffold to the highly charged bisphosphonates, which are less likely to pass bacterial membranes.

## Abstract 2

The crystal structures of farnesyl diphosphate synthase from the pathogen *Pseudomonas aeruginosa* in complex with substrates and inhibitors have been determined. The study reveals the presence of an allosteric binding pocket also in bacterial enzymes that, similar to the human enzyme, could be the target for the design of specific and potent inhibitors.

## 1. Introduction   

Isoprenoid biosynthesis is a ubiquitous biological process that is found in all kingdoms of life. Isoprenoids constitute one of the largest group of metabolites on Earth (>30 000 compounds) and are involved in a diverse range of biochemical functions that are often critical for the survival of the organism (Lange *et al.*, 2000 ▶). In recent years isoprenoid biosynthesis has received considerable attention, in part owing to the discovery of the nonmevalonate pathway found in plant species and many pathogenic bacteria (Rohmer, 1999 ▶). This metabolic route towards isoprenoids differs completely from the more established mevalonate pathway, and several of the enzymes involved have become attractive targets for antibacterial drug-discovery initiatives as they are not found in humans (Oldfield, 2010 ▶).

**Figure 1:**
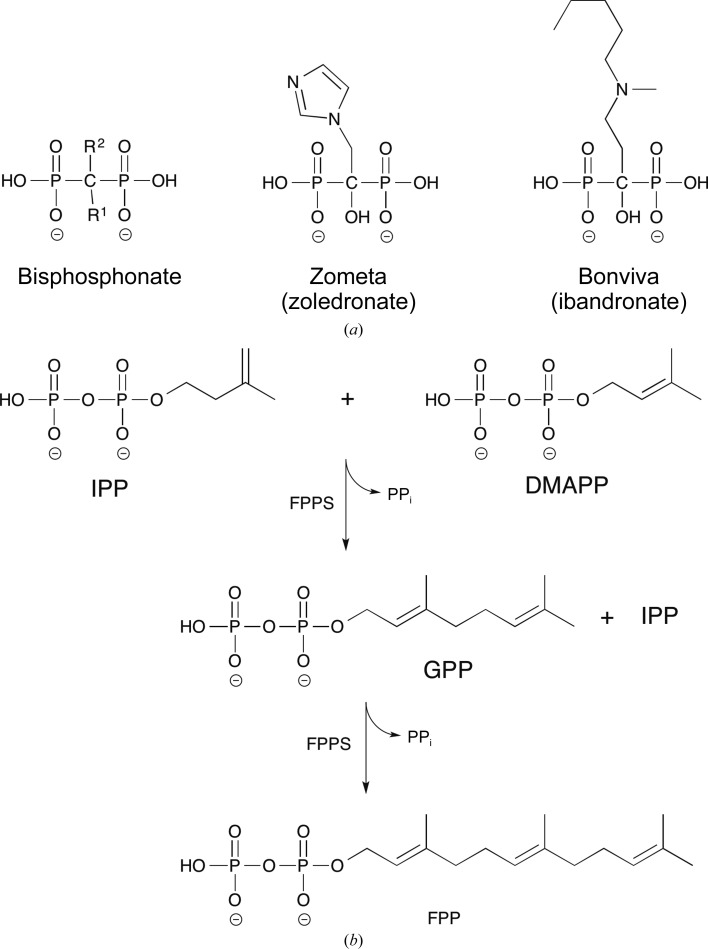
(*a*) Chemical structures of bisphosphonates. Nitrogen-containing bisphosphonates such as zoledronate and ibandronate have a hydroxyl group in the *R* ^1^ position and nitrogen-containing *R* ^2^ side chains. (*b*) Farnesyl pyrophosphate synthase-catalyzed condensation reactions. Isopentenyl diphosphate (IPP) first reacts with dimethylallyl diphosphate (DMAPP) to form geranyl diphosphate (GPP), releasing pyrophosphate (PP_i_). GPP then reacts with IPP to form farnesyl pyrophosphate (FPP) and free PP_i_.

Farnesyl pyrophosphate synthase (FPPS; EC 2.5.1.10) is a promising antibiotic drug target as it represents the point of convergence of these two pathways (Dhar *et al.*, 2013 ▶). FPPS has been listed as indispensible for the survival for a number of pathogenic bacteria (Luo *et al.*, 2014 ▶). The enzyme belongs to a large group of prenyltransferases responsible for the principal bond-forming reactions of isoprenoid biosynthesis (Poulter *et al.*, 1978 ▶; Song & Poulter, 1994 ▶). The mevalonate and nonmevalonate pathways both produce isopentenyl pyrophosphate (IPP), which along with its isomer dimethyl­allyl diphosphate (DMAPP) is converted through a ‘head-to-tail’ condensation reaction to form geranyl diphosphate (GPP) by FPPS (Fig. 1 ▶). While prenyltransferases do exhibit some range in selectivity of product length, FPPS tends not to synthesize a prenyl chain longer than the 15-carbon isoprenoid chain of farnesyl pyrophosphate (FPP; Tarshis *et al.*, 1996 ▶; Dhar *et al.*, 2013 ▶). FPP is involved in protein prenylation and in the formation of triterpenes, both of which are important for cell survival (Oldfield, 2010 ▶).

Since the 1980s, human FPPS has been the target enzyme of a very successful group of inhibitors, the nitrogen-containing bisphosphonates (N-BPs; Fig. 1 ▶ *a*). As stable analogues of pyrophosphate (PP_i_), the bisphosphonate (BP) core consists of a P–C–P backbone with geminal *R* ^1^ (typically a hydroxyl) and *R* ^2^ groups. Early work on BPs focused on their capacity to inhibit the action of osteoclasts, cells that are responsible for bone resorption in mammals. These drugs have been in clinical use for treatment of a variety of bone diseases such as Paget’s disease of bone, osteoporosis and skeletal complications of malignancy (Russell *et al.*, 2008 ▶). The N-BPs represent a refined subset of the BPs which are several orders of magnitude more potent than their predecessors (Russell, 2011 ▶). They feature primary or tertiary N atom(s) in their *R* ^2^ group, the importance of which was not known until the late 1990s (over a decade after their first discovery) when the general biochemical mode of action of the N-BPs was elucidated (Van Beek *et al.*, 1999 ▶; Dunford *et al.*, 2001 ▶).

Owing to a high affinity for hydroxyapatite, all BPs efficiently accumulate in bone tissue. Specifically, N-BPs target FPPS in osteoclasts, thereby reducing bone destruction by disrupting cell signalling through inhibition of protein prenylation (Rogers, 2003 ▶). It is this tissue specificity that has been a strength of this class of drugs with regard to their efficacy and their safety in clinical medicine. However, N-BPs are a versatile drug class, with pre-clinical evidence of anticancer activity through direct interaction with tumour cells (Clézardin & Massaia, 2010 ▶). Furthermore, they have also been used to inhibit the growth of pathogenic eukaryotic microorganisms (Montalvetti *et al.*, 2003 ▶; Gabelli *et al.*, 2006 ▶; Huang *et al.*, 2010 ▶) by targeting the FPPSs of these organisms.

The first structural context of N-BP binding was the crystal structure of *Escherichia coli* FPPS (EcFPPS) with risedronate bound in the active site (Hosfield *et al.*, 2004 ▶). Subsequently, in 2006 two groups published structures of the human enzyme in complex with a range of N-BPs (Kavanagh *et al.*, 2006 ▶; Rondeau *et al.*, 2006 ▶). N-BP–FPPS complex structures have also been reported for the eukaryotic microorganisms *Trypanosoma cruzi* (Gabelli *et al.*, 2006 ▶) and *Leishmania major* (Aripirala *et al.*, 2014 ▶). In all cases, the *R* ^2^ side chain was observed to bind in the hydrophobic cleft that normally accommodates the growing isoprenoid chain, while the P–C–P head was coordinated by Mg^2+^ ions and two motifs containing conserved aspartic acid residues. Binding of N-BPs induced a closed conformation of the enzymes. Furthermore, the binding mode of the cyclic N atoms supports the hypothesis that N-BPs mimic a carbocation reaction intermediate (Kavanagh *et al.*, 2006 ▶). More recently, in an effort to address the issue that N-BPs are too specific for bone minerals for effective anticancer treatment, Green and coworkers identified the first potent non-N-BP inhibitors of FPPS (Jahnke *et al.*, 2010 ▶). Crystallographic studies revealed a novel mode of binding of these inhibitors to an allosteric site in human FPPS, providing an additional binding pocket for the development of a new range of FPPS inhibitors (Lindert *et al.*, 2013 ▶; De Schutter *et al.*, 2014 ▶).

The locus PA4043 in the genome of the human-pathogenic bacterium *Pseudomonas aeruginosa* PA01 contains an open reading frame that had been annotated as coding for a farnesyl pyrophosphate synthase (PaFPPS). Here, we provide biochemical evidence that this gene indeed encodes a farnesyl pyrophos­phate synthase and report the crystal structures of the un­liganded enzyme and its complex with the bound substrate geranyl pyrophosphate. We show that the enzyme is strongly inhibited by ibandronate (Bonviva) and zoledronate (Zometa), drugs that are in clinical use, and we have determined the crystal structure of PaFPPS with ibandronate to 1.85 Å resolution. We further report ligand-bound structures of the enzyme derived from a fragment-library screen that show binding of these small molecules in the allosteric binding site as seen in human FPPS. Importantly, the study shows that this allosteric site is also found in FPPS from prokaryotic organisms and that it is significantly less conserved than the active site between human and bacterial FPPSs. It thus emphasizes this ligand-binding pocket as a potential target for strong-binding FPPS inhibitors that might be developed into antibacterial drugs.

## 2. Materials and methods   

### 2.1. Enzyme production and purification   

The open reading frame encoding the putative FPPS was amplified from the *P. aeruginosa* PA01 genome by PCR and the resulting fragment was inserted into the pET-28-based vector pNIC28-Bsa4 (Savitsky *et al.*, 2010 ▶). This construct was transformed into *E. coli* BL21 (DE3) cells and expressed in 1 l cultures of LB with 30 µg ml^−1^ kanamycin at 20°C until an OD_600_ of 0.6 was reached before induction using 0.1 m*M* IPTG. Cells were harvested 24 h after induction. The construct had a fused N-terminal six-histidine tag with a TEV protease cleavage site. Recombinant PaFPPS was purified using Ni–NTA affinity resin (Qiagen) in batch mode, followed by His-tag cleavage using TEV protease. The solution containing the cleaved enzyme was re-run on Ni–NTA resin and the flowthrough was concentrated to 1 ml and transferred onto an Superdex 200 gel-filtration column (GE Healthcare, Uppsala, Sweden) equilibrated with 25 m*M* Tris–HCl pH 8, 150 m*M* NaCl. Fractions containing PaFPPS were collected, concentrated to 25 mg ml^−1^, flash-frozen in liquid nitrogen and stored at −80°C.

### 2.2. Crystallization and structure determination   

Crystals of PaFPPS were grown using sitting-drop vapour diffusion in CrystalClear P strips (Douglas Instruments). Native crystals were grown using 20% PEG 3350, 0.2 *M* NaF, 0.1 *M* bis-tris propane pH 6.5 as the mother liquor. A protein concentration of 25 mg ml^−1^ and 2:1 µl drops (protein:mother liquor) were used. The well volumes were always 60 µl. Crystals intended for preparation of complexes were grown in one of two similar conditions. Condition 1 consisted of a 2:1 µl drop ratio with 0.2 *M* MgCl_2_, 20% PEG 6000, 0.1 *M* Tris pH 8. Condition 2 consisted of a 2:1 µl drop ratio with 0.15 *M* MgCl_2_, 20% PEG 8000, 0.1 *M* Tris pH 8. For the ibandronate complex, a tablet of the drug (Roche) was ground up and dissolved in deionized water. The soluble fraction was used as a 100 m*M* stock solution based on the reported mass of the drug in each tablet. Enzyme crystals grown in condition 1 were transferred into a fresh drop with the same composition containing 5 m*M* ibandronate and were soaked for 20 h. For fragment complexes, native enzyme at 25 mg ml^−1^ was pre-incubated for 1 h with 20 m*M* KM10833 or 10 m*M* SPB02696 before setup of the crystallization experiments. For the geranyl pyrophosphate (GPP) complex, a condition 1 drop containing native crystals was supplemented with 0.5 µl GPP solution (2.74 m*M* in methanol), yielding final concentrations of 0.46 m*M* GPP and 16.7% methanol. The crystals were incubated for 1 h before flash-cooling in liquid nitrogen. For the GPP/SPB02696 complex, 10 µl of enzyme (25 mg ml^−1^) was co-crystallized (condition 2, with 15% glycerol) with 1 µl each of 2.74 m*M* GPP and 50 m*M* SPB02696, resulting in a solution consisting of 20 mg ml^−1^ FPPS, 0.228 m*M* GPP, 4.16 m*M* SPB02696, 10% methanol, 1% DMSO before crystallization.

Crystals of the native enzyme and the complexes with SPB02696 and GPP/SPB02696 were harvested directly from the drops and flash-cooled, while crystals containing KM10833 were first transferred to a reservoir solution supplemented with 15% PEG 1500 before cooling. All X-ray data sets were collected on beamlines ID14-1 and ID14-4 at the European Synchrotron Radiation Facility (ESRF). In all cases, diffraction data were collected from a single cooled crystal at 100 K. Data were indexed and integrated using *MOSFLM* (Battye *et al.*, 2011 ▶) except for those of the GPP complex, which were processed by *XDS* (Kabsch, 2010 ▶). Scaling of the data sets was performed using either *SCALA* (Evans, 2006 ▶) or *AIMLESS* from the *CCP*4 program suite (Winn *et al.*, 2011 ▶). X-ray diffraction statistics are summarized in Table 1 ▶.

The PaFPPS structure was solved by molecular replacement using *Phaser* (McCoy *et al.*, 2007 ▶) and the coordinates of the homologue from *P. fluorescens* Pf-5 (∼76% identity; PDB entry 3lji; New York SGX Research Center for Structural Genomics, unpublished work) as a search model. One polypeptide chain was used as the search model, with the conserved amino-acid side chains retained, whereas non­conserved residues were replaced by alanine side chains. The crystal asymmetric unit contains a dimer related by a twofold noncrystallographic symmetry axis. Initially, the structure was modelled to 2.2 Å resolution with *ARP*/*wARP* (Joosten *et al.*, 2008 ▶) and the resolution was then extended to 1.5 Å using data collected from a crystal from an unsuccessful soaking experiment with a fragment molecule from the Maybridge library (2.5 m*M* CC35801, 5% DMSO). All model building and refinement was carried out by iterative cycles of *REFMAC* (Murshudov *et al.*, 2011 ▶) and *Coot* (Emsley *et al.*, 2010 ▶). Local NCS restraints were used throughout the refinement and rotamers were modelled where indicated by the electron-density maps. The overall quality of the model was assessed using *MolProbity* (Chen *et al.*, 2010 ▶). Model-refinement statistics are reported in Table 2 ▶.

The structures of the enzyme–ligand complexes were determined using the coordinates of the refined PaFPPS structure. Refinement consisted of iterative rounds of model building using *Coot* and refinement runs with *REFMAC*. In the final refinement runs ligand and water molecules were built into the difference densities in the 2*F*_o_ − *F*_c_ electron-density maps. The final model statistics are given in Table 2 ▶. Crystallo­graphic data have been deposited in the Protein Data Bank with accession codes 3zcd (un­liganded FPPS), 4umj (FPPS–ibandronate), 3zl6 (FPPS–KM10833), 3zmb (FPPS–SPB02696), 3zmc (FPPS–GPP) and 3zou (FPPS–GPP–SPB02696).

### 2.3. Fragment-based screening   

Differential scanning fluorimetry was used to monitor the thermal denaturation of FPPS and to screen for stabilizing ligands in the Maybridge 500 fragment library (http://www.maybridge.com). The 25 µl assay mixtures consisted of 5 µg PA4043 and Maybridge 500 library compounds (dissolved in DMSO) at 10 m*M* concentration with 100 m*M* Tris–HCl pH 8.0 as the assay buffer. After 20 min incubation of the samples with the compounds, 0.5 µl SYPRO Orange (Sigma) reporter dye was added to 0.1%(*v*/*v*). The fluorescence intensity data were monitored as described previously (Ericsson *et al.*, 2006 ▶) and the melting-point (*T*_m_) values were determined from sigmoidal fits. All measurements were carried out in triplicate. In order to filter out solvent effects, control wells with 2% DMSO were run in parallel.

### 2.4. Enzyme assay   

Reagents were sourced from Sigma–Aldrich unless otherwise stated. FPPS activity was determined by coupling the PaFPPS-catalysed condensation reaction of GPP with isopentyl pyrophosphate (IPP), forming farnesyl pyrophos­phate (FPP) and pyrophosphate, with yeast pyrophosphatase (PPase; Roche). The PPase converted the pyrophosphate to orthophosphate, which was detected through a modified malachite green (MG) assay as described previously (Baykov *et al.*, 1988 ▶). In brief, a MG colour reagent stock was made by slowly adding 60 ml concentrated H_2_SO_4_ to 300 ml water; the solution was cooled to room temperature and then supplemented with 0.44 g MG. A daily MG working solution (MGWS) was composed of 10 parts colour reagent, 2.5 parts 7.5% (NH_4_)_2_MoO_4_ and 0.2 parts 11% Tween 20. A standard reaction involved 0.1 *M* Tris–HCl pH 8, 5 m*M* MgCl_2_, 0.05 U ml^−1^ PPase, 20 µ*M* GPP, 20 µ*M* IPP and 25 µ*M* PaFPPS. The reaction series was initiated by the addition of PaFPPS and allowed to react for 10 min at room temperature before being stopped by the addition of one part MGWS to four parts reaction solution. After 20 min of incubation, the absorbance at 620 nm was recorded. A 96-well plate-based assay system was used with a reaction volume of 100 µl plus 25 µl of MGWS to yield a total well volume of 125 µl for spectrometric reading on a BioTek plate reader at 620 nm.

### 2.5. Enzyme-inhibition assay   

The inhibitory effects of ibandronate and zoledronate on PaFPPS activity were investigated using the assay described above. Ibandronate was obtained from Enamine (catalogue No. EN300-119513) and zoledronate was obtained from Sigma. Assays were carried out in 0.2 ml total volumes at 22°C in triplicate. The reaction mixtures consisted of 100 m*M* Tris–HCl pH 8.0, 5 m*M* MgCl_2_, 20 µ*M* geranyl pyrophosphate, 20 µ*M* isopentenyl pyrophosphate, 0.05 U ml^−1^ pyrophos­phatase, 25 n*M* PaFPPS. Ibandronate was used in a 0.0076–150 µ*M* concentration range (three dilution steps, ten different concentrations) in the reaction. 30 µl samples were taken at 2, 5, 10, 15 and 20 min time points and developed by the addition of 12 µl MG reagent in low-volume microtitre plates (Corning 3994) to quantify the phosphate ions produced in the coupled assay. The absorbance at 620 nm was recorded by a BioTek plate reader. The reaction rates were derived from linear fits to the absorbance data and the activity values were normalized to the rate of the non-inhibited reaction. IC_50_ values were derived from sigmoidal fits using *Origin*.

The IC_50_ values for zoledronate were determined in a similar way using an inhibitor range of 0.00025–5.0 µ*M* and an enzyme concentration of 12.5 n*M*. None of the inhibitors affected the activity of the coupling enzyme (yeast PPase) or the derivatization of phosphate ions by the malachite green reagent in the concentration range used.

## 3. Results   

### 3.1. Locus PA4043 in *P. aeruginosa* PAO1 encodes a farnesyl pyrophosphate synthase   

PA4043 encodes a protein of 295 amino acids that was recombinantly produced in *E. coli* and purified to homogeneity as judged by SDS–PAGE. The purified recombinant protein was analyzed by ESI-MS and the detected mass of 34 017.5 Da exactly matched the mass calculated from the amino-acid sequence, including the His tag.

PA4043 is annotated as a farnesyl pyrophosphate synthase. In order to provide biochemical evidence for this enzymatic activity, we employed a pyrophosphatase coupled activity assay that is also amenable to high-throughput screening. FPPS catalyses successive condensation reactions of IPP with its isomer dimethylallyl pyrophosphate (DMAPP) and GPP to form FPP (Fig. 1 ▶ *b*). The first step is the formation of GPP from IPP and DMAPP. In a second step GPP reacts with IPP again to form FPP. The developed assay couples the FPPS activity to pyrophosphatase (EC 3.6.1.1), producing free orthophosphate from the released product pyrophosphate. The ortho­phosphate is then detected by the malachite green assay. To ensure control of the stoichiometry of pyrophos­phate release, GPP rather than DMAPP was chosen as a substrate, along with IPP, thus limiting the reaction to the second step. Under this regime, the condensation of one molecule of IPP with one of GPP releases one free pyrophosphate ion, which in turn yields two orthophosphate ions through the action of PPase. Indeed, it was observed that no reaction took place without both IPP and GPP being present, meaning that there was no detectable spontaneous isomerization of IPP to DMAPP which could have interfered with the stoichiometry of the assay reaction (data not shown). Recombinant PaFPPS showed a catalytic activity of 0.49 s^−1^, which is comparable to the activity of human FPPS (0.42 s^−1^; Kavanagh *et al.*, 2006 ▶). Although strictly speaking the assay only demonstrates the second reaction, the conversion of GPP and IPP to FPP, the data nevertheless provide clear evidence of PA4043 being a farnesyl pyrophosphate synthase.

**Figure 2:**
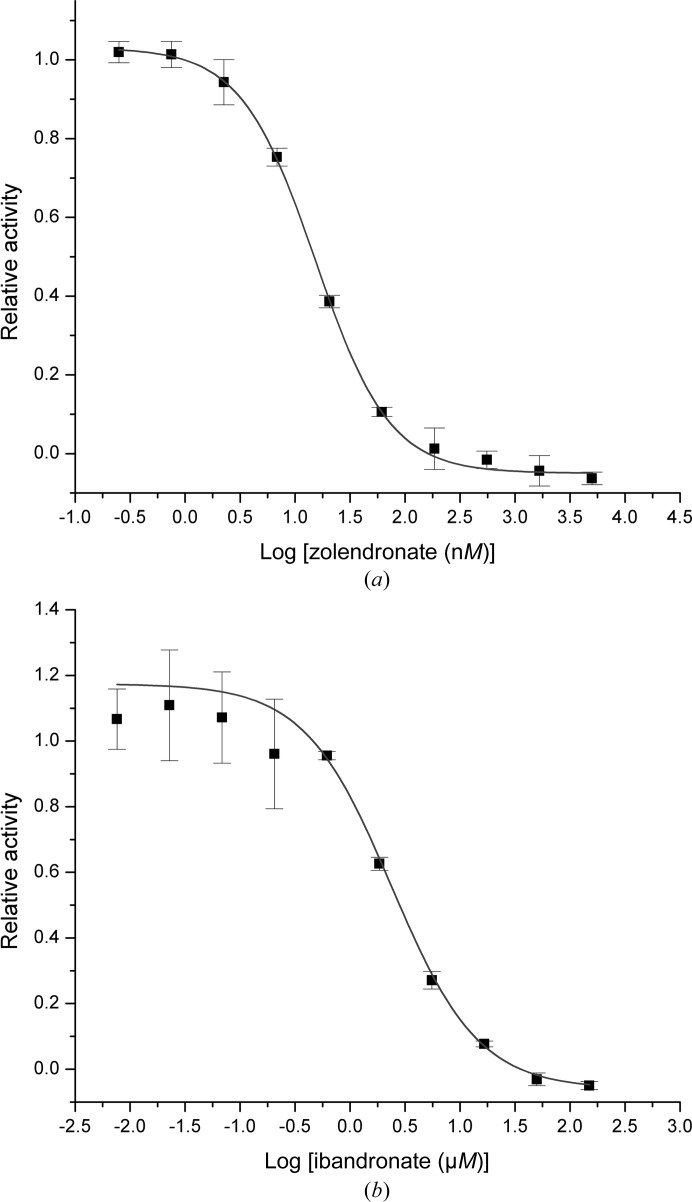
The inhibitory effect of zoledronate (*a*) and ibandronate (*b*) on farnesyl pyrophosphate synthase from *P. aeruginosa*. The dose-response curves resulted in IC_50_ values of 15.2 n*M* and 2.7 µ*M*, respectively.

This assignment is further strengthened by the influence of well known FPPS inhibitors that are in clinical use: ibandronate and zoledronate. Both compounds were shown to inhibit PaFPPS in a concentration-dependent manner, with an IC_50_ value of 2.7 ± 0.4 µ*M* for ibandronate, while zoledronate is a considerably stronger inhibitor with an IC_50_ value of 15.2 ± 0.7 n*M* (Fig. 2 ▶). This parallels the relative effects of these drugs on FPPS from human and *T. brucei*, with zoledronate being one of the strongest N-BP inhibitors (Russell *et al.*, 2008 ▶). Zoledronate inhibits the human enzyme with an IC_50_ of 4.1 n*M* (Kavanagh *et al.*, 2006 ▶) and the trypanosomal enzyme with an IC_50_ of 400 n*M* (Yin *et al.*, 2006 ▶). The corresponding IC_50_ values for ibandronate are 20 n*M* (Dunford *et al.*, 2001 ▶) and 3.1 µ*M* (Yin *et al.*, 2006 ▶), respectively.

### 3.2. Overall structure of PaFPPS   

**Figure 3:**
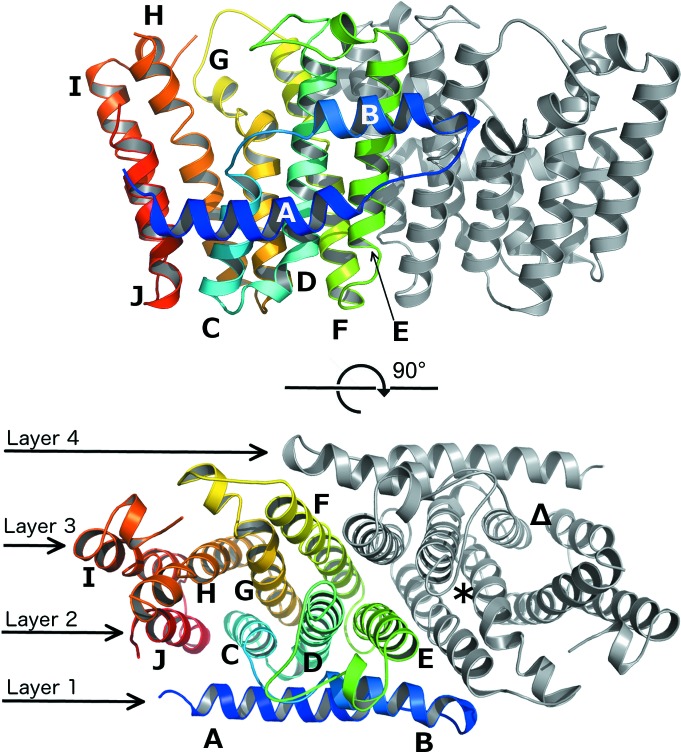
Upper panel: cartoon representation of the PaFPPS homodimer with chain *A* coloured from blue to red from the N-terminus to the C-terminus. Chain *B* is shown in grey. Helices are labelled A–J. Lower panel: a 90° rotation about a horizontal axis to show the layered topology of the PaFPPS dimer. In chain *B* (grey) the S1 binding site for inhibitors such as ibandronate and the allosteric site are indicated by an asterisk (S1 site) and a triangle (allosteric site).

The structure of recombinant FPPS from *P. aeruginosa* PA01 was determined and refined to a resolution of 1.55 Å (*R* = 15.1%, *R*_free_ = 20.4%; Table 2 ▶). The polypeptide chain is well defined in the electron-density map, with the notable exception of the mobile loop region between helices H and I (loop-HI; Fig. 3 ▶) consisting of 27 residues (Ala227–Gly254; amino-acid numbering is for PaFPPS unless otherwise indicated). This loop region is completely disordered in all PaFPPS structures reported here. The asymmetric unit contains a homodimer, with a twofold axis relating the two subunits (Fig. 3 ▶). The overall topology of this α-helical structure is consistent with the isopentyl pyrophosphate synthase fold (SCOP ID 48577; Murzin *et al.*, 1995 ▶) characterized in this class of enzymes by a two-helix N-terminal hairpin (helices A and B; Fig. 3 ▶) lying orthogonal to a central eight-helix antiparallel bundle (Tarshis *et al.*, 1994 ▶; Gabelli *et al.*, 2006 ▶; Huang *et al.*, 2010 ▶). In the homodimer, the hairpin and bundle helices collectively form four continuous layers. Each subunit features its own active-site cleft located between helices C, G, H and J. The N-terminal hairpin helices (A and B) form key intersubunit interactions with helix E of the second subunit, although helices E and F form the bulk of the dimer interface. The buried surface area at the dimer interface is 6490 Å^2^ as determined by *PISA* (Krissinel & Henrick, 2007 ▶). The structure of the PaFPPS subunit shows little variation from the relatively conserved core FPPS fold (Tarshis *et al.*, 1994 ▶), with an r.m.s.d. of 2.7 Å to the evolutionary divergent human homologue HsFPPS (Rondeau *et al.*, 2006 ▶; PDB entry 2f7m; 233 equivalent C^α^ atoms, 23.1% amino-acid sequence identity) and 0.7 Å to its closest relative in the PDB, the putative geranyltransferase from *P. fluorescens* (PDB entry 3lji; 263 equivalent C^α^ atoms, 74.3% amino-acid sequence identity).

### 3.3. Binding of GPP to PaFPPS   

Two structures of PaFPPS with bound GPP were determined in the course of this study: a ternary complex with GPP and Mg^2+^ and a quaternary complex with GPP, Mg^2+^ and a small molecule derived from screening of a fragment-based library (Table 2 ▶). The overall structures of the enzyme sub­units in these two complexes are very similar to the structure of the unliganded enzyme (r.m.s.d. values of around 0.3 Å) and no large-scale conformational changes have been observed. Note that the lid region, residues Ala227–Gly254, is also disordered in these two structures. In the PaFPPS complex GPP is well defined in both subunits, whereas in the quaternary complex GPP is only bound to subunit *A*. However, the binding mode and interactions of GPP with protein residues and Mg^2+^ ions are identical in the two complexes.

**Figure 4:**
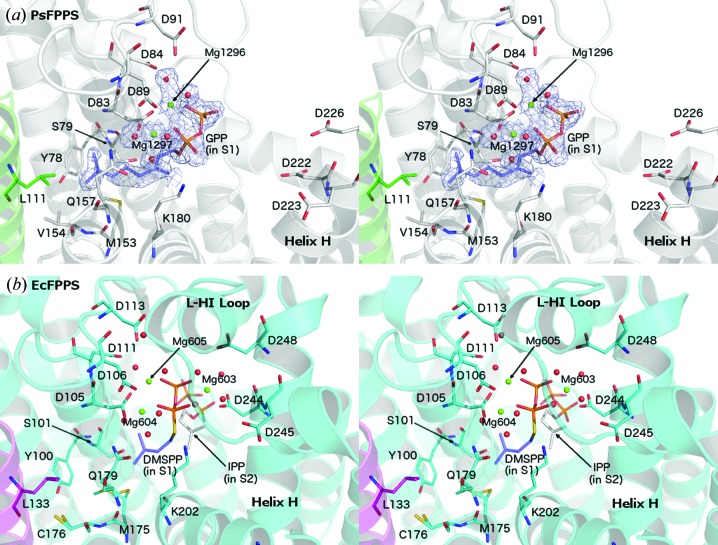
Comparison of farnesyl pyrophosphate synthase complexes from *P. aeruginosa* (PaFPPS; this study) and *E. coli* (EcFPPS; PDB entry 1rqi; Hosfield *et al.*, 2004 ▶). (*a*) Stereo representation of part of subunit *A* of PaFPPS in complex with GPP and Mg^2+^. Electron density (*F*_o_ − *F*_c_ OMIT map contoured at 1.0σ) is shown around the bound ligands. Asp83, Asp84 and Asp89 form part of motif I, while Asp222, Asp223 and Asp226 form part of motif II. (*b*) Stereoview of the active site of EcFPPS in complex with DMSPP (S1 site), IPP (S2 site) and Mg^2+^. Residue numbering is specific to the respective enzymes.

GPP binds in the allylic binding site (S1), with the pyrophosphate moiety anchored *via* two Mg^2+^ ions that are bound to the conserved aspartate-rich sequence motif I DD*XXXX*D (Hosfield *et al.*, 2004 ▶; residues Asp83–Asp89 in PaFPPS) located at the end of helix D (Fig. 4 ▶ *a*). Together with phosphate O atoms, water molecules and residues Asp83 and Asp89, the two metal ions (Mg1296 and Mg1297 in subunit *A*; throughout the text we will use the ligand numbering as in the deposited PDB files) are bound with octahedral coordination geometry to the enzyme active site. The additional aspartate residues Asp84 from motif I and Asp91 interact indirectly with Mg1296 through water molecules. The hydrophobic tail of GPP extends into a predominantly hydrophobic pocket lined by Tyr78, Ser79, Met153, Val154, Gln157 and Lys180. Leu111 of the second subunit (helix E) also contributes to forming the end of this pocket. The location of GPP and this part of the ligand–enzyme interactions are similar to those described for the noncleavable DMAPP substrate analogue DMSPP (dimethylallyl *S*-thiolodiphosphate) in *E. coli* FPPS (EcFPPS; Hosfield *et al.*, 2004 ▶), except that the longer isoprenyl chain of GPP penetrates deeper into this hydrophobic pocket (Fig. 4 ▶ *b*).

An additional Mg^2+^ binding site is formed by motif II DD*XX*D (Asp222–Asp226) located at the end of helix H and preceding the mobile loop-HI lid. In the *B* subunits of the PaFPPS ternary complex this site is occupied by a Mg^2+^ ion bound with octahedral coordination geometry to Asp223 and the surrounding water molecules, about 6.5 Å away from the pyrophosphate group of GPP. In other complexes of farnesyl pyrophosphate synthases with ligands bound in the S1 site (Hosfield *et al.*, 2004 ▶; Kavanagh *et al.*, 2006 ▶; Rondeau *et al.*, 2006 ▶), this metal ion is shifted towards the pyrophosphate group of the ligand owing to a rigid-body movement of helices H and I and participates in the formation of a trinuclear metal centre (Mg603 in Fig. 4 ▶ *b*) which is implicated in the catalytic mechanism of this enzyme family (Hosfield *et al.*, 2004 ▶). Formation of this trinuclear centre in PaFPPS would require a similar conformational change, including a shift of helix H and the ordering of the mobile lid region, all resulting in closure of the S1 site. For human FPPS (HsFPPS) this closure has been shown to complete the formation of site S2 and allow binding of IPP (Rondeau *et al.*, 2006 ▶). In the structure of the PaFPPS–GPP complex the active site is more open and these conformational changes have not occurred upon binding of the substrate to the S1 site.

### 3.4. The PaFPPS–ibandronate complex reveals multiple inhibitor-binding sites   

In order to investigate the structural basis of inhibition of PaFPPS by N-BPs, we determined the structure of the enzyme in complex with ibandronate (Bonviva) to 1.85 Å resolution (Tables 1 ▶ and 2 ▶). The structure of the enzyme polypeptide chains in this inhibitor complex is similar to the structure of the unliganded enzyme and other enzyme complexes, with r.m.s.d. values in the range 0.2–0.3 Å for the *A* and *B* subunits. We note that also in the ibandronate complex the active site is in the open conformation and the lid region is disordered.

**Figure 5:**
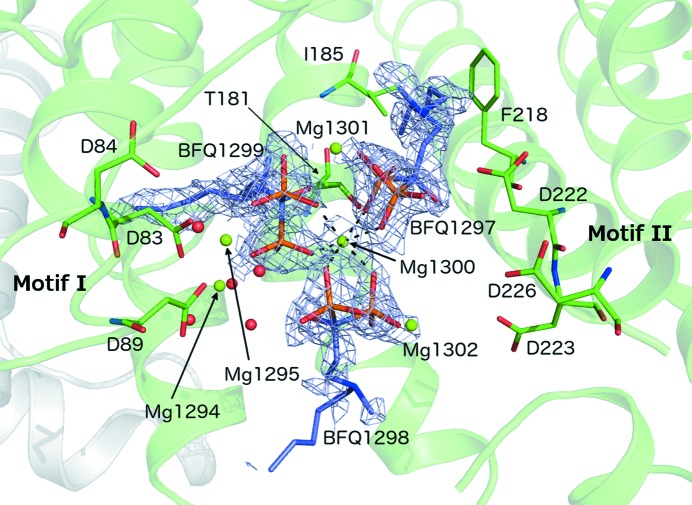
Clustering of three ibandronate molecules (BFQ) with associated Mg^2+^ ions in the active site of PaFPPS (chain *A*). Electron density (*F*_o_ − *F*_c_ OMIT map at 1.0σ) is shown around the ibandronate molecules. Motif I (DD*XXXX*D) is represented by Asp83, Asp84 and Asp89. Motif II (DD*XX*D) is represented by Asp222, Asp223 and Asp226. One molecule of ibandronate (BFQ1299) is located in the S1 binding site, similar to the binding mode of GPP seen in Fig. 4 ▶(*a*).

In the following, we describe the mode of inhibitor binding and enzyme–ligand interactions for subunit *A*, as the ligand molecules are best defined in electron density in this subunit. Three ibandronate molecules were bound in the active site in this subunit (Fig. 5 ▶). Molecule BFQ1299 is located in site S1, the common binding site for the DMAPP/GPP substrates and N-BP inhibitors in other FPPSs (Hosfield *et al.*, 2004 ▶; Kavanagh *et al.*, 2006 ▶; Zhang *et al.*, 2009 ▶; Huang *et al.*, 2010 ▶). The hydrophobic tail of this ibandronate molecule extends into the same pocket as the isoprenyl tail of GPP, which is lined by residues Tyr78, Ser79, Leu111, Met153, Val154, Gln157 and Lys180. Like the diphosphate group of GPP (see above), the bisphosphonate group is anchored to the protein *via* two Mg^2+^ ions (denoted Mg1294 and Mg1295 in this complex), which in turn are bound to the enzyme by two aspartate residues from motif I: Asp81 and Asp83. The two Mg^2+^ ions show an octahedral coordination geometry, with O atoms from the two aspartate residues bridging the two metal ions, two O atoms from the bisphosphonate group and water molecules. A third Mg^2+^ ion (Mg1300) is bound at the opposite site of the bisphosphonate group and completes the trinuclear metal site seen in FPPS–ligand complexes from other species (Hosfield *et al.*, 2004 ▶; Huang *et al.*, 2010 ▶; Kavanagh *et al.*, 2006 ▶; Zhang *et al.*, 2009 ▶). However, owing to the absence of the hinge motion, motif II is not close enough to provide ligands to this metal ion. In place of these stabilizing interactions from motif II, Mg1300 together with two additional Mg^2+^ ions (Mg1301 and Mg1302) provides the anchoring point of two additional ibandronate molecules that are bound at the active site (Fig. 5 ▶). The hydrophobic tail of molecule BFQ1297 extends into a rather open pocket lined by Thr181, Ile185, Phe218 and Asp222, with the side chain of the latter interacting with the tertiary amino group of the inhibitor. This pocket is part of the IPP binding site (S2) and interacts with the isoprenyl tail of the second substrate (Hosfield *et al.*, 2004 ▶).

The third ibandronate molecule, BFQ1298, is anchored through its bisphosphonate head group *via* Mg1300 to the head groups of the other two ibandronate molecules. The hydrophobic tail of this molecule extends into the bulk solution and is not defined in electron density beyond the tertiary N atom. In subunit *B* there is no electron density for a third ibandronate molecule and therefore it was not modelled.

A superimposition of the structures of the PaFPPS–Mg^2+^–ibandronate complex and the HsFPPS–Zn^2+^–ibandronate complex (PDB entry 2f94; Rondeau *et al.*, 2006 ▶) resulted in an r.m.s.d. of 2.9 Å based on 269 equivalent C^α^ atoms. The major structural difference is the lack of lid closure in the *P. aeruginosa* enzyme. Furthermore, helix F of PaFPPS, which contributes to the formation of the S1 site, carries a two-residue insertion (Ser150 and Ala151) in the middle of the helix which results in a local break of the helical conformation. Ibandronate molecule BFQ1299 in the PaFPPS complex binds to the canonical S1 binding site as in the complex with the human enzyme, with many of the enzyme–ligand interactions being conserved in the two enzymes. In HsFPPS the bound inhibitor was observed in two slightly different conformations (Rondeau *et al.*, 2006 ▶). However, the electron-density maps for the PaFPPS–ibandronate complex did not indicate multiple conformations. The positions of the metal ions Mg1294 and Mg1295 and their counterparts in the HsFPPS–Zn^2+^–ibandronate complex, Zn1001 and Zn1003, are very similar and the metal–ligand interactions are conserved in the two enzymes. However, the enzyme–metal interactions for the third metal ion, Mg1300 and Zn1002, respectively, differ. In both cases the metal ions bind to the bisphosphonate group of the inhibitor at the S1 site. Zn1002 further interacts with two aspartic acid side chains from motif II. In the PaFPPS–Mg^2+^–ibandronate complex the corresponding Mg^2+^ ion is too far from residues of motif II owing to the lack of lid closure. Instead, this metal ion also interacts with the bisphosphonate groups from the two other ibandronate molecules bound in the active site (Fig. 5 ▶).

### 3.5. Ligands derived from fragment-based screening bind at different sites compared with N-BP inhibitors   

**Figure 6:**
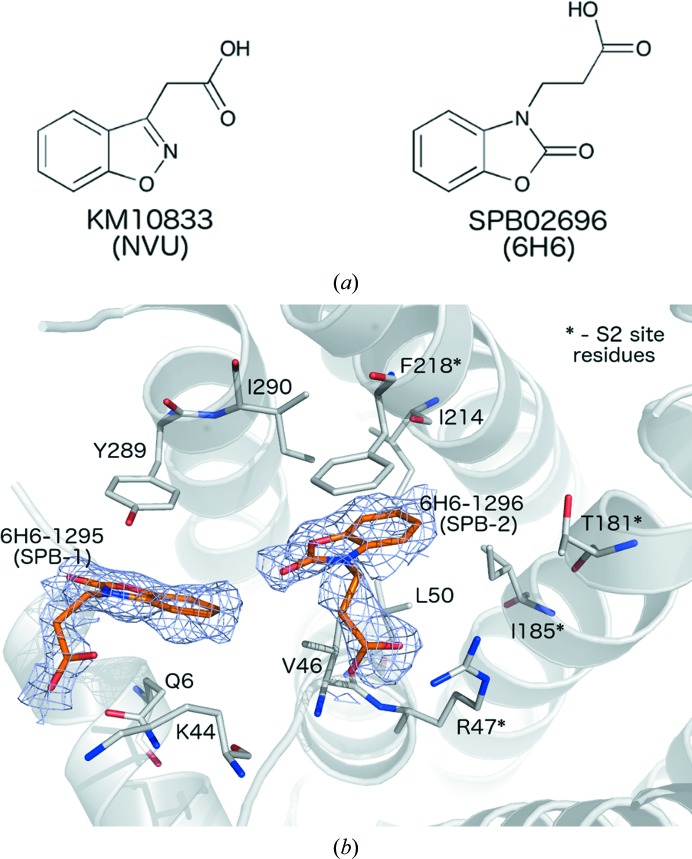
(*a*) Chemical structures of the fragments KM10833 (left) and SPB02696 (right) identified to bind to the allosteric site of PaFPPS in this study. (*b*) Structure of the PaFPPS–SPB02696 complex. Sites SPB-1 and SPB-2 are occupied by two molecules of SPB02696 (6-H6-1295 and 6-H6-1296, respectively) and their proximities to the S2 site (formed by the residues indicated with asterisks) are shown. An *F*_o_ − *F*_c_ OMIT map covering each ligand is shown in blue (contour level at 1.0σ).

Thermal shift analysis using differential scanning fluorimetry was employed to screen a fragment library (Maybridge 500 Fragment Library) to identify compounds stabilizing PaFPPS. Of the 500 fragments of this library, only two molecules, KM10833 [2-(benzo[*d*]isoxazol-3-yl)acetic acid] and SPB02696 {3-[2-hydrozybenzo[*d*]oxazol-3(2*H*)-yl]propanoic acid} (Fig. 6 ▶ *a*), caused significant shifts of the thermal melting temperature, by 4.2 and 2.8°C, respectively. However, enzymatic assays using these compounds at a concentration of 10 m*M* did not show any significant inhibition, most likely owing to the weak binding constants of these small fragments. We were able to determine crystal structures of these hits with PaFPPS, including a ternary enzyme–GPP–SPB02696 complex (Tables 1 ▶ and 2 ▶). The overall structures of the polypeptide chains in these complexes are very similar to those of other PaFPPS structures determined in the course of this study (r.m.s.d. values typically in the range 0.2–0.4 Å) and represent the open structure of the enzyme with the lid region disordered.

In subunit *A* of the binary complexes of PaFPPS with KM10833 or SPB02696 (Fig. 6 ▶ *a*), two binding sites in a hydrophobic pocket adjacent to the active site were found. For convenience we will term these binding sites SPB-1 and SPB-2 (Fig. 6 ▶ *b*). Both fragment hits bind at the same locations in this pocket and we describe the details of the enzyme–ligand interactions for fragment hit SPB02696 (termed 6H6 in the deposited coordinate file). Site SPB-2 is located deeper in this pocket, in the vicinity of the binding site of the IPP substrate. The ligand is bound in a hydrophobic pocket lined by Val46, Leu50, Ile185, Ile214, Phe218 and Ile290. The ring system is sandwiched between the side chains of Val46 and Phe218, and the carboxyl group of the ligand forms a hydrogen bond to the side chain of Arg47 (Fig. 6 ▶ *b*). The second molecule is bound about 6 Å away at the outer fringes of this pocket in site SPB-1, with the ring system sandwiched between the side chains of Tyr289 and Gln6 (Fig. 6 ▶ *b*). The carboxyl group extends into the bulk solution, but is located in the vicinity of the side chain of the surface residue Lys44. In the *B* subunit we only observe binding of SPB02696 to site SPB-1. In the case of KM10833 no binding to subunit *B* was observed. In the ternary PaFPPS–GPP–fragment complex, the fragments only bound to site SPB-1.

**Figure 7:**
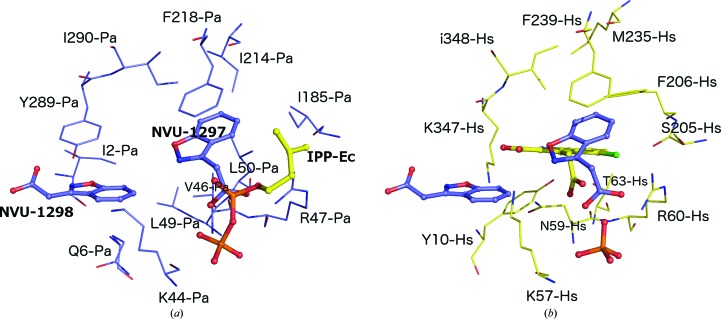
Comparison of the allosteric binding sites in (*a*) PaFPPS (blue C atoms) and (*b*) HsFPPS (yellow C atoms). (*a*) The PaFPPS allosteric site with the two bound KM10833 molecules (NVU-1298 and NVU-1297). The location of the IPP binding site is illustrated by superimposition of the EcFPPS–IPP complex (PDB entry 1rqi). (*b*) Three fragment ligands bound to FPPS are shown in ball-and-stick representation. One ligand (C atoms coloured yellow) is a representative example found to bind in the allosteric binding site of HsFPPS with an accompanying sulfate ion (PDB entry 3n5h; Jahnke *et al.*, 2010 ▶). The other two (blue-coloured C atoms; NVU-1298/NVU-1297) are the superposed KM10833 from the PaFPPS complex shown in (*a*) (PDB entry 3zl6). Residues from each structure forming direct contacts are labelled.

This binding pocket has recently also been observed in human FPPS. In an effort to identify a new class of FPPS inhibitors that moved away from the classic bisphosphonate moiety of the N-BP pharmacophore, Green and coworkers observed a novel pattern of ligand binding and inhibition for the human enzyme which is identical to the binding pocket for the fragment hits described here. This allosteric binding site was discovered from a fragment-based screen and was successfully exploited to develop novel and potent non-N-BP inhibitors of human FPPS (Jahnke *et al.*, 2010 ▶; Lindert *et al.*, 2013 ▶; De Schutter *et al.*, 2014 ▶). The finding that this pocket is also present in bacterial enzymes is of particular interest as it could provide the basis for the development of specific antibacterial compounds. Residues lining this alternative binding pocket and located within a 8 Å sphere from the bound fragments show a lower level of conservation between the human and *P. aeruginosa* FPPS enzymes (30% amino-acid identity) when compared with the level of conservation seen for the active site (including sites S1 and S2) itself (60% amino-acid identity) and might thus be better suited for specific inhibitor design. Among the major differences in the composition of the allosteric binding pocket are substitutions of the hydrophilic residues Asn59, Thr63, Ser205 and Lys347 in HsFPPS by their counterparts Val46, Leu50, Ile185 and Tyr289 in the bacterial enzyme (Fig. 7 ▶). Furthermore, helix G, which participates in the formation of the allosteric binding site, displays structural differences in the two enzymes. In HsFPPS this helix contains a single-residue insertion at position 206, resulting in a local distortion of the helical conformation and substantial local differences in the topology of the allosteric site. Residue Phe206 points into the allosteric site and interacts with bound ligands in HsFPPS. In addition, the position of Tyr10 in HsFPPS in the pocket is replaced by the side chain of Leu49 in PaFPPS owing to structural differences at the N-termini of the two enzymes. Overall, these substitutions contribute to structural differences in the allosteric binding site and to a more hydrophobic character of this site in PaFPPS compared with its counterpart in the human enzyme.

## 4. Discussion   

Locus PA4043 had been annotated to encode a putative farnesyl pyrophosphate synthase. In this study, we have shown that recombinant expression of this open reading frame in *E. coli* yields a soluble enzyme that indeed displays activity as a farnesyl pyrophosphate synthase. Furthermore, the enzyme is strongly inhibited by drugs that are currently in clinical use as inhibitors of the human homologue. The enzymatic activity, the inhibition profile and the three-dimensional structure thus provide experimental evidence for the assignment of PA4043 as encoding a farnesyl pyrophosphate synthase.

**Figure 8:**
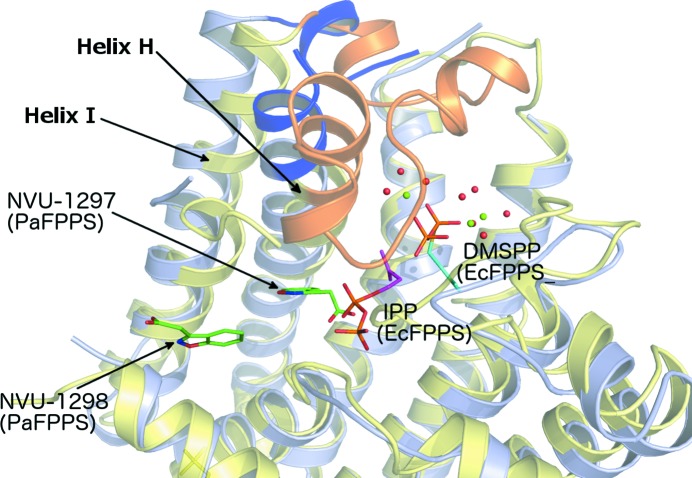
Cartoon representation of the superimposition of the structures of EcFPPS (yellow; PDB entry 1rqi) and PaFPPS (grey). The loop-HI region that is disordered in PaFPPS (highlighted by the blue-coloured flanking regions of helix H and part of the loop itself) is ordered in the closed conformation in EcFPPS (orange). DMSPP and IPP are bound as ligands in the structure of EcFPPS and represent the allylic binding site (S1) and the IPP binding site (S2) of this class of enzyme. NVU-1298 and NVU-1297 represent the SPB-1 and SPB-2 sites of the allosteric binding pocket in PaFPPS, respectively. The two bound KM10833 molecules are shown in green.

In both bacterial (Hosfield *et al.*, 2004 ▶) and eukaryotic (Rondeau *et al.*, 2006 ▶; Kavanagh *et al.*, 2006 ▶; Zhang *et al.*, 2009 ▶) farnesyl pyrophosphate synthases, binding of ligands bearing either pyrophosphate groups or bisphosphonate groups in the S1 site leads to conformational changes that can be described as a small rotation of the C-terminal part of the enzyme, in particular helices H and I, and a disorder–order transition of the loop connecting these two helices (Fig. 8 ▶). The conformational changes result in completion of the active site, because helix H carries motif II involved in the binding of the third Mg^2+^ ion of the trinuclear metal site. Furthermore, the lid region folds over the active site and leads to partial occlusion of the allylic substrate-binding site from the bulk solution. In the human enzyme binding of IPP in site S2 leads to a fully closed conformation as the last C-terminal basic residues fold over the IPP binding site (Kavanagh *et al.*, 2006 ▶). In the course of this study we have determined a range of binary and ternary complexes of PaFPPS with ligands occupying the S1, SPB-1 and SPB-2 binding sites. In all cases, however, the enzyme is observed in the open conformation, *i.e.* the active site is not fully assembled and poised for catalysis owing to a lack of movement of helix H/I and lid closure. We suggest that crystal lattice forces also select for the open conformation for the enzyme–substrate complex, thus preventing the formation of a catalytically relevant complex. Analysis of crystal packing of all structures shows that helices H (residues 201–228) and I (residues 255–274) and loop residues 247–254 pack against neighbouring molecules in the crystal lattice. These interactions might prevent the rigid-body rotation of the C-terminal helices that is required for active-site assembly upon substrate binding.

A somewhat surprising observation was the presence of multiple ibandronate molecules in the active site of the PaFPPS–inhibitor complex. One of the inhibitor molecules is bound in the allylic S1 site as observed in N-BP complexes of FPPS from other species. In PaFPPS two (in subunit *A*) or one (in subunit *B*) additional ibandronate molecules are bound to the active site, located in the cleft between the core of the subunit and the C-terminal residues that move to close the active site. At first glance they appear to act as wedges to prevent these conformational changes. However, we consider the binding of these additional inhibitor molecules to be a consequence of the open conformation of the enzyme in the crystal lattice rather than a novel, alternative mode of inhibition by N-BPs in PaFPPS.

Nitrogen-containing bisphosphonate inhibitors of FPPS are characterized by a highly charged head group which makes these compounds not very promising as antibacterial agents owing to their difficulties in passing through the bacterial cell wall. The discovery in the human enzyme of a novel inhibitor-binding site and the development of new lead compounds of more hydrophobic character, with less affinity for bone, suggested that FPPS inhibitors might be developed that target diseases other than bone-related diseases (Jahnke *et al.*, 2010 ▶; De Schutter *et al.*, 2014 ▶; Liu *et al.*, 2014 ▶). Through the screening of a small fragment library, we could demonstrate that this binding pocket also exists in the bacterial enzymes and might be exploited for the development of new lead candidates for antibiotics. Given the fact that this site is less conserved than the active site in bacterial *versus* human FPPS, the design of specific inhibitors of bacterial enzymes might be possible.

Finally, we note a systematic difference in the occupancy of ligands in the two subunits of PaFPPS. It appears not to be related to soaking of ligands into the active site, *i.e.* differences in accessibility of the two active sites in the asymmetric unit, as this finding also holds for the PaFPPS–GPP–fragment complex obtained by co-crystallization. It is possible that these observations reflect cooperative behaviour in the dimeric enzyme with respect to substrate/ligand binding, but this remains to be corroborated by further experimental studies.

In summary, this work provides the first functional and structural insights into FPPS from the opportunistic pathogen *P. aeruginosa*. We show that the enzyme is strongly inhibited by N-BPs that are presently in clinical use and identify a hitherto unexploited binding site in the bacterial enzyme that, similar to the human enzyme, might be a target for the design of specific and potent inhibitors.

## Supplementary Material

PDB reference: farnesyl pyrophosphate synthase, complex with SPB02696, 3zmb

PDB reference: complex with KM10833, 3zl6

PDB reference: complex with GPP, 3zmc

PDB reference: complex with GPP and SPB02696, 3zou

PDB reference: complex with ibrandronate, 4umj

## Figures and Tables

**Table 1:** Data-collection statistics for PaFPPS and enzymeligand complexes Values in parentheses are for the outer shell.

Data set	Native	Ibandronate	KM10833	SPB02696	GPP	GPP + SPB02696
PDB code	3zcd	4umj	3zl6	3zmb	3zmc	3zou
Space group	*C*222_1_	*C*222_1_	*C*222_1_	*C*222_1_	*C*222_1_	*C*222_1_
Unit-cell parameters						
*a* ()	84.2	85.3	85.2	85.6	86.0	85.0
*b* ()	98.5	98.6	98.8	98.8	98.8	98.6
*c* ()	131.1	131.3	131.6	131.5	130.6	130.5
Wavelength ()	0.9334	0.9801	1.0032	1.0032	0.9763	0.9334
Resolution ()	1.55	1.85	1.85	1.90	1.87	1.55
*R*_merge_ (%)	10.3 (35.9)	10.1 (52.7)	11.7 (72.2)	11.9 (65.8)	10.9 (64.9)	6.1 (53.9)
Total reflections	287186	200587	212082	163467	227673	327621
Unique reflections	78501	46925	47364	43864	46143	77089
Mean *I*/(*I*)	7.2 (3.1)	7.9 (1.8)	8.2 (1.9)	7.1 (1.9)	9.2 (1.7)	13.1 (1.9)
Completeness (%)	99.5 (99.4)	98.8 (94.5)	99.6 (100)	99.3 (100)	99.9 (100)	96.9 (75.8)
Multiplicity	3.7 (3.4)	4.3 (2.9)	4.5 (4.4)	3.7 (3.9)	4.9 (5.0)	4.2 (2.8)
Wilson *B* (^2^)	14.6	19.7	19.1	16.9	15.5	13.2

**Table 2:** Refinement and model statistics for PaFPPS and enzymeligand complexes

Data set	Native	Ibandronate	KM10833	SPB02696	GPP	GPP + SPB02696
Resolution ()	1.55	1.85	1.85	1.90	1.87	1.55
*R*_work_/*R*_free_	0.151/0.204	0.202/0.242	0.175/0.222	0.188/0.241	0.205/0.251	0.162/0.195
No. of atoms						
Total	4684	4742	4560	4793	4553	4808
Protein	4125	4068	4094	4134	4084	4166
Ligand/ion	0	104	67	50	53	72
Water	559	570	429	609	416	570
R.m.s.d.s						
Bond lengths ()	0.015	0.016	0.018	0.016	0.018	0.018
Bond angles ()	1.608	1.764	1.775	1.662	1.864	1.894
*B* factors (^2^)						
Overall	22.4	26.1	22.2	22.3	20.4	18.8
Protein	21.7	25.3	21.7	21.5	20.0	18.0
Ligands		26.5	49.6	40.5	24.0	30.2
Waters	36.9	34.1	27.8	31.1	28.3	29.4
Ramachandran plot						
Favoured (%)	99.1	99.1	98.9	98.9	98.9	99.1
Outliers (%)	0.0	0.0	0.2	0.0	0.0	0.0
